# Essay on Cholera

**Published:** 1867-01

**Authors:** N. Wright

**Affiliations:** Chatham, Ill.


### ARTICLE V.

### ESSAY ON CHOLERA.

By N. WRIGHT, M.D., Chatham, Ill.

Read before the Illinois State Medical Society, June 6, 1866.

The almost certain reäppearance of that formidable disease, Asiatic Cholera, amongst us the ensuing year, should claim the especial attention of medical men, whose duty it is to gather around them all the information that is possible to be obtained concerning the various theories of its causes, pathology, and treatment.

It would seem presumptuous in me to attempt to enlighten you, gentlemen, upon this already thread-bare theme; still the vast importance of the subject, and the intense interest that is manifested by the public mind, demand that the little we may know, or the theories we may entertain, should become the common property of the profession, as well as the community at large, whose protectors and guardians, in matters of health, we are. They look to us in "this the hour of their distress," and we should be prepared by our science and skill to calm the public fear, and thus remove one of the great, if not the greatest, predisposing causes of the frightful epidemic. We should investigate the subject in all its various phases, and when we do meet the disease, meet it calmly, with a mind well prepared by sound pathology and therapeutics, early to battle with the monster and gain the victory, thus showing to the community that the confidence reposed in us, as conservators of the public weal, is not misplaced. Let us be no cowards, but, armed with science and experience, go forth and conquer even the fell destroyer cholera.

I would define cholera, as a disease that is characterized by profound disorder of every vital function—of innervation, circulation, respiration, and secretion. It is attended with more or less prostration and lassitude; with a suspension of all the secretions; a feeling of cold; frightfully sensible to the touch; the breath and tongue of almost icy feel; a voice so peculiar as to entitle it to the *sobriquet* of "vox cholerica;" with a leaden color of the skin and mucous membranes, wherever perceptible; shrunken features and sodden extremities; cramps of the abdomen and extremities; a pulse quite small and feeble, often imperceptible; respiration accompanied with frequent sighing, and quite difficult—all of these attended with a profuse flux, both *per orem et anum*, of an enormous quantity of sero-albuminous fluid, deficient or entirely wanting in the normal secretions, heavily loaded with mucous epithelium, which has been mistaken for an altered secretion—all of these generally ending in death by asphyxia.

The appearances presented upon dissection are all much the same, with perhaps more uniformity than in most other diseases. The only discrepancy can be easily accounted for, could we but know the particular stage of the disease in which the subject died. That discrepancy is found in the condition of the lungs, sometimes pale and exsanguious, and at others engorged and turgid with blood. The first, or anæmic condition, I have found when death took place in the early stage of collapse; and the hyperæmic condition, when the collapse has passed away and reäction, either in whole or part, has been established. The chief constant and conspicuous pathological phenomenon that is apparent in every case, is the changed condition of the blood, deprived of its serum, which has passed away in the enormous discharges and left a dark, coagulated mass accumulated in the right cavities of the heart, the vena cava, the portal and emulgent veins. The arteries are, for the most part empty, as, also, is the left side of the heart. The blood thus found, coagulates entirely, without leaving a single drop of serum; heavily loaded with carbon and coloring matter; deficient in the saline ingredients; without a particle of the free alkalies contained in healthy blood. Urea sometimes exists in the blood, where the secretion of urine has not been partially reëstablished previous to death. The mucous surfaces are covered with a substance almost precisely like the rice-water discharges vomited and purged during life. There is mucous epithelium still adherent to the surfaces of the membrane. The liver is filled to overflowing with dark blood, arising, no doubt, from both the obstruction of the central organ of circulation and the loss of fluidity of the blood itself. The bladder is invariably found empty and closed firmly upon itself, affording a diagnostic mark between cholera-morbus and cholera. The spleen is found often enormously distended, and, in fact, not a single abdominal or thoracic organ can be found unmarked with vascular rupture or turgescence of black blood.

Let us, for a few moments, contemplate the changes we find in the various organs from their normal condition, together with the offices they perform in the living machine, and I think we must all come to the conviction that the essence of the disease consists in morbid action of the secreting organs, produced by some poison, diffused throughout the blood, having a special direction and influence upon the nerves of organic life, or, in other words, upon the great sympathetic or ganglionic system of nerves whose duty it is to preside over the three great operations of life, to wit:—circulation, respiration, and secretion.

In the cold stage of our ordinary intermittent, in the rigor of congestive fever, and in the symptoms of some remittents, we have a few of the symptoms of this disease, though they are but in miniature and of the milder form. We also behold some of its phenomena in our ordinary endemic cholera-infantum—the same persistent nausea; the same albuminous discharge. And, still again, we meet with a closer resemblance in our intermittents, when they assume the malignant, or, as it is often called, the congestive form.

I have seen, and, doubtless, most of you have seen, cases of malignant chills which were almost identical with cholera—the same coldness of the surface and sodden appearance of the extremities; the same shrunken, pinched countenance; even the "vox cholerica," with the small and often imperceptible pulse; attended too, by vomiting, purging, and cramps. It was the close resemblance of one of these cases, years ago, when intermittents were of a more malignant character than now, that led me to investigate the relations that exist between cholera and miasmatic diseases; not that they are precisely alike, but there is a striking analogy between them in some of their symptoms, particularly the organs most affected. The locality too, which produces cholera in its most frightful forms, is identically the same as of miasmatic diseases. India, on the banks of the Ganges, the cradle of the scourge; the delta of the Nile; the valleys of the Mississippi and the Rio Grande; and, in short, wherever you find alluvial deposits and malarious regions, there you will find favorable localities for its propagation and foci from which it disseminates itself.

I would not wish to be understood as classing cholera with diseases of miasmatic origin, simply because they are rife in the same regions that cholera has ravaged and still continues to devastate; nor yet from the strong resemblance that cholera has in some of its earlier symptoms to this class of diseases; but I do think that there is a strong family resemblance between them, and that all the phenomena of the disease clearly indicate that both are of that class of disease which first shows itself in its effects by the derangement of the nerves of organic life.

To elucidate, in part, the pathology of the disease, I would call your attention to the fact, that the ònus of the morbid impressions is first felt by the chylopoietic viscera, which are supplied with nerves from this system. When we consider the physiological purpose of the ganglionic system of nerves, and that all vital actions are produced through their influence; that they are distributed to the heart, lungs, liver, spleen, and kidneys, as well as to all the viscera contained in the thoracic and splanchnic cavities, and that these organs feel the touch of the disease almost simultaneously, we are compelled to admit that it is upon these nerves that the disease is hurled.

The doctrine that malarious diseases were derangements of the ganglionic system of nerves, is no new theory; but, until quite recently, the fact, as such I may now call it, has not been demonstrated. Prof. J. H. SALISBURY, in his experiments, as related in the *American Journal of Medical Sciences*, shows that the essence of malarial poisoning consists of minute cells of an algid type, closely resembling palmella that are produced under favorable circumstances of heat and moisture, inhaled into the lungs, thence through the blood; and these affect the vital functions of the nerves of organic life; change the normal condition of the secreting organs, and thus produce malarial poisoning. Such may be, and quite likely is, the manner of the working of cholera, at least there is a striking resemblance between the operations of the two classes of poisons—affecting the same organs in much the same manner, through the same means, though with a different degree of intensity.

From a careful survey of the various epidemics, as narrated by careful observers, and from no inconsiderable experience in the disease, I am fully satisfied that it can only be communicated through and by means of matter vomited or purged that may have an immediate effect upon systems prepared to receive them; or they may act remotely upon the putrid products of decay, made up of decomposing animal and vegetable matter, in our large cities; hence, the intensity and diffusion of cholera is in an inverse ratio to the sanitary condition of the place. Atmospheric causes have, doubtless, much to do, as remote agents, in spreading the disease, still they are unable to produce it without the aid of local inoculation of existing filth and decomposing matter by cholera poison from some one having the disease. It travels no faster than it is carried, and stringent quarantine and strict sanitary measures will do much to avert, if not to exclude, it from any place.

If the foregoing views of the origin and pathology of this disease are correct, and the more I read and reason upon the subject, the more I am convinced they are, the plan of treatment must be simple and plain, but the means by which to accomplish it *hoc opus, hic labor est.*

The obvious pathological changes, as we have previously stated, are in the blood, and an impaired or subverted innervation of the ganglionic system of nerves presiding over the functions of organic life, that are distributed to the heart, lungs, and viscera contained in the abdomen.

With these changes constantly in view, our efforts will be, first, to sustain the vital powers; then, to arouse and change the impaired action or non-acsion of the splanchnic nerves; then to arouse and restore the suspended secretions and compel the various organs *revocare gradum.*

All remedies may be, and indeed often are, abused or misused; and stimulants, in this our day, seem to be in some danger of abuse, as it is the fashion to stimulate for everything. I would wish to use them so as to avoid Scylla on one side, and Charybdis on the other. The scripture tells us to "give strong drink to him that is ready to perish, and wine to him that is of feeble heart," and I know of no disease that "fills the bill" of the wise man so completely full as the one now under consideration. Active and diffusible stimulants must be freely used to overcome the extreme prostration and bring about the so much desired reäction. We must restore the lost equilibrium of the circulation and innervation, arouse the capillary circulation of the skin and mucous membranes, as well as the organs contained in the abdomen; reëstablish the biliary and urinary secretions, and even when these are accomplished, our work is hardly begun. The vast quantities of effete matters that are retained in the blood, the result of suspended secretions, must be eliminated before we can feel assured that our patient *superasque evadere ad auras.* When we look for a moment at the enormous amount of secretion in a healthy adult, the saliva, the gastric juice, the pancreatic fluid, the bile, the urine, the cutaneous exhalations, and carbonic acid thrown off by the lungs and think that these are all retained in the system, why do we wonder that death takes place so soon as it often does in cholera? Why not rather wonder that the patient survives so long?

One great point in the pathology of cholera, which has attracted more attention than all others, is the anæmic condition of the left side of the heart and arteries, and the excessive hyperæmia of the right side of the heart and veins. Some late observers and writers have attributed this to spasm, or rather, as they have called it, *tetanic* spasm of the arteries and capillaries, particularly of the branches of the pulmonic arteries, that has closed these vessels and thus prevents the transmission of the blood through the lungs, and, consequently, no blood enters the lungs to be there oxygenated and passed to the left side of the heart, and again to pass its rounds in the system. At first view, there would seem to be some plausibility in this hypothesis. The success that blood-letting and nauseants, and even emetics, and, more recently, chloroform, have met with by their known powers of relaxing spasm, and thus (as is claimed by the supporters of this tetanic spasm theory) permitting the transmission of the blood through these closed capillaries, I admit looks very reasonable, if the obstacle to the circulation be of the nature of tetanic spasm. But if, on the other hand, a want or absence of tone in the capillary vessels and branches of the pulmonic arteries, arising from deficient innervation of the nerves that govern the circulation be the cause that obstructs the blood and prevents its entrance into the lungs, there to receive the vivifying element that fits it for sustaining vital actions, if this be the cause, as I believe, it is evident that these means can afford but temporary relief and, in the end, but hasten the fatal result. The reason that seems to me perfectly satisfactory for the stoppage of the blood in the right side of the heart and in the pulmonary artery, is a want of nervous power that should be imparted by the sympathetic, which system is overpowered or stricken down by the poison of the disease. With this view of the causes that produce the unequal circulation, our attention should be directed to arousing the energies of the nervous system, particularly the ganglionic nerves.

The best means to accomplish this with, in my hands, in many cases of malignant chills, has been large and oft-repeated dry cups applied to the epigastric region, for beneath this region lies the great epigastric or solar plexus of the sympathetic system, from which plexus radiates the nerves that supply nearly all the viscera in the abdomen. I need not enumerate the various ganglia and their distribution, but if there is one point in the human body that is, or can be called, the centre of organic life, the solar plexus is that point. The shock imparted to the nervous system through the nerves radiating from this plexus by a large cup applied and repeated over this region, has saved patients of mine, suffering with malignant intermittents that I do not believe could have been saved by any other means in my power; the nervous system was aroused by this means, and stimulants and tonics completed the work. Strong rubefacients should be assiduously applied along the entire length of the spine while cramps or spasms continue, and friction with the hands and flour of mustard or capsicum where-ever they appear. External heat, in the form of hot baths, whenever they can be had, with mustard freely infused, must be used for the purpose of keeping up the temperature of the body and arousing the capillary circulation of the skin.

An excellent, and in the country, in the absence of a bathing tub, a very convenient method of administering external heat is by means of ears of maize boiled for some time in water, and placed, while hot, around the patient in bed, or what is called a corn sweat; mustard sinapisms to the wrists and inside of the thighs and ankles; hot pediluvia, increased with red pepper or capsicum; and a flannel dipped out of hot water and sprinkled quickly with spts. terebinth., applied hot to the abdomen; and injections of warm water per anum, as warm as you dare to use, to the amount of two or three pints, together with warm bed-clothing and the recumbent position. All of these should be persevered in as adjuvants to the medical treatment.

I am well aware that treating symptoms is not a very sound or rational practice, but we have high authority for combating or avoiding the tendency to death, and we are justified in our efforts to alleviate the pain our patient is suffering while we are awaiting the action of medicines that may restore him to health. Vomiting is one of the symptoms that distress and harass the patient, and at the same time prevents the retention of medicines we may have administered to overcome the disease. Cold or ice-water is very grateful to the sufferer. and often it seems to check the vomiting. Ice-cold sparkling wines, as catawba or champaign, often are the best drinks that can be given our patients. Creosote, in doses of three or four drops in a little cinnamon water, with about half an ounce of gum Arabic emulsion, will sometimes check this distressing symptom. Mint juleps and the common effervescing soda-water, mixed with ice, is an excellent means for allaying the distressing vomiting, and at the same time it alleviates the tormenting thirst.

Opium, we all know, in small doses, frequently administered, acts as a stimulant, and, at the same time, will allay the excessive irritability and pain that accompanies the early stages of the disease. It, however, is a remedy that must be used with great caution and carefully watched, that narcotism is not produced, and it should be altogether withdrawn when the consecutive fever stage springs up.

Ipecachuana, in small doses, will be found of great assistance, by its effects upon the skin, and combined with camphor, which will do much to sustain the enfeebled nerves, and at the same will do all that we can do with medicine to relieve the frequent spasm.

Calomel must not be forgotten, to arouse the dormant secretions, especially of the biliary organs; but the enormous doses that have been given in this disease are but a wanton waste of a useful medicine. The quantities of each will vary with the peculiar characteristics of the epidemic and with different cases of the same epidemic, as well as the intervals of time in which they should be administered.

| | | | |
|---|---|---|---|
| ℞. | Hyd. Sub Murias, ------ | grs.xvj. | ℈ij. |
| | G. Camph. pulv., ----- | grs.xviij. | ʒss. |
| | Pulv. Ipecach., ---------- | grs.xij. | grs.xviij. |
| | Pulv. Opii, ------------ | grs.xij. | grs.xiv. |

M. Divide into 24 pills.

From the strong resemblance of many things in cholera to miasmatic diseases, and the well-established efficacy of quinine in the latter, it would seem well worthy a place among our remedies in this disease, and many have used it with much satisfaction. Oxygen gas, could it be easily obtained, would be a powerful assistant before the collapse stage of the disease.

This is but an imperfect outline of the treatment I would propose for the early stage of the disease, and often the judicious treatment of this stage will obviate the necessity of treating the more advanced stages and severer forms.

But should the disease progress in spite of all our efforts, and bad cases generally will, and extreme collapse ensue, shall we give up in despair? Cold effusions, in the form of shower baths, applied so as to produce as great a nervous shock as possible, have been known to save the patient when in *articulo mortis*. Opiates can rarely be used now, and all our efforts must be used in every possible way to reänimate the almost dead body—diligent friction, after the cold shower bath; hot blankets and strong irritants more assiduously applied to the spine and abdomen; and the stimulants, both alcoholic and diffusible, should now be carried almost to the heroic—these should be tried, but, I confess, with little hope of success in this stage of the disease.

The consecutive fever that follows the recovery from cholera is often of as much importance as the disease itself. Local inflammation, arising from the suspended secretions in the various organs, will demand our attention, with appropriate means for their removal, which will best be understood by the appearance of the case itself. Local abstraction of blood, with counter-irritants, and even blisters, together with appropriate remedies for restoring the secretions that have been suspended, these, with a light, nutritious diet, will generally restore our patients to health again.

*Resume.*—1. Cholera very closely resembles diseases of miasmatic origin, as well in the choice of its location as in the identity and manner of the organs affected, and in the success that the same treatment has with both diseases.

2. That both miasmatic diseases and cholera are essentially manifested in derangement of all the secretions dependent upon deficient circulation of the blood, caused by want of innervation of the nerves of organic life belonging to the ganglionic system that are stricken down by the poison of the disease.

3. That the first and greatest object in the treatment of this disease, is to arouse the energy of these nerves by some sudden shock, so that they will supply the proper stimulus to the heart and arteries sufficient to keep up the circulation which may be much assisted by keeping the temperature of the body up to, or above, the normal condition.

4. That the proper and judicious use of stimulants, nervous, alcoholic, and diffusible, with other means mentioned above, to arouse the dormant secretions after the nervous system has been aroused, afford us much encouragement in the treatment of this disease.

